# Human granulocyte colony stimulating factor (hG-CSF): cloning, overexpression, purification and characterization

**DOI:** 10.1186/1475-2859-7-13

**Published:** 2008-04-04

**Authors:** Ana LS Vanz, Gaby Renard, Mario S Palma, Jocelei M Chies, Sérgio L Dalmora, Luiz A Basso, Diógenes S Santos

**Affiliations:** 1 Programa de Pós-Graduação em Biologia Celular e Molecular, PUCRS. Av. Ipiranga, 6690, Partenon, Porto Alegre, 90610000, Brazil; 2 Centro de Pesquisas em Biologia Molecular e Funcional, Instituto de Pesquisas Biomédicas, PUCRS. Av. Ipiranga, 6681, Tecnopuc, Prédio 92A, Partenon, Porto Alegre, 90619900, Brazil; 3 Quatro G Pesquisa e Desenvolvimento LTDA. Av. Ipiranga, 6681, Tecnopuc, Prédio 92A, Partenon, Porto Alegre, 90619900, Brazil; 4 Laboratório de Biologia Estrutural e Zooquímica, Centro de Estudos de Insetos Sociais, Departamento de Biologia, Instituto de Biociências, Universidade Estadual Paulista, Rio Claro, 13506-900, Brazil; 5 Departmento de Farmácia Industrial, Centro de Ciências da Saúde, Universidade Federal de Santa Maria, Santa Maria, 97105-900, Brazil

## Abstract

**Background:**

Biopharmaceutical drugs are mainly recombinant proteins produced by biotechnological tools. The patents of many biopharmaceuticals have expired, and biosimilars are thus currently being developed. Human granulocyte colony stimulating factor (hG-CSF) is a hematopoietic cytokine that acts on cells of the neutrophil lineage causing proliferation and differentiation of committed precursor cells and activation of mature neutrophils. Recombinant hG-CSF has been produced in genetically engineered *Escherichia coli *(Filgrastim) and successfully used to treat cancer patients suffering from chemotherapy-induced neutropenia. Filgrastim is a 175 amino acid protein, containing an extra N-terminal methionine, which is needed for expression in *E. coli*. Here we describe a simple and low-cost process that is amenable to scaling-up for the production and purification of homogeneous and active recombinant hG-CSF expressed in *E. coli *cells.

**Results:**

Here we describe cloning of the human granulocyte colony-stimulating factor coding DNA sequence, protein expression in *E. coli *BL21(DE3) host cells in the absence of isopropyl-β-D-thiogalactopyranoside (IPTG) induction, efficient isolation and solubilization of inclusion bodies by a multi-step washing procedure, and a purification protocol using a single cationic exchange column. Characterization of homogeneous rhG-CSF by size exclusion and reverse phase chromatography showed similar yields to the standard. The immunoassay and N-terminal sequencing confirmed the identity of rhG-CSF. The biological activity assay, *in vivo*, showed an equivalent biological effect (109.4%) to the standard reference rhG-CSF. The homogeneous rhG-CSF protein yield was 3.2 mg of bioactive protein per liter of cell culture.

**Conclusion:**

The recombinant protein expression in the absence of IPTG induction is advantageous since cost is reduced, and the protein purification protocol using a single chromatographic step should reduce cost even further for large scale production. The physicochemical, immunological and biological analyses showed that this protocol can be useful to develop therapeutic bioproducts. In summary, the combination of different experimental strategies presented here allowed an efficient and cost-effective protocol for rhG-CSF production. These data may be of interest to biopharmaceutical companies interested in developing biosimilars and healthcare community.

## Background

Biopharmaceuticals are medicinal products comprising biotechnology-derived recombinant proteins as active substances, according to the European Agency for the Evaluation of Medicinal Products [1]. Biopharmaceuticals have revolutionized the treatment of many diseases. Some of the biopharmaceutical patents have already expired, thereby allowing production of follow-on or biosimilar products. Public and private sectors of a number of countries have been encouraged to share staff, funding and facilities to increase technology transfers between universities and the industry [2]. It is due to an emerging multibillion-dollar market for companies willing to produce biosimilar versions of these products and to potential savings for healthcare payers and consumers that represents a driver of demand for biosimilars. Filgrastim (recombinant human granulocyte colony stimulating factor, rhG-CSF), produced by Amgen, had its patent expired in 2006. This biopharmaceutical generated global sales of $5.6 billion (June 2005 to June 2006) and its market in Europe and USA has the potential to generate sales of approximately $605 million in 2010 [3].

The granulocyte colony stimulating factor (G-CSF) is central to neutrophil-based immune defenses due to its regulatory role in the growth, differentiation, survival, and activation of neutrophils and their precursors [4]. Cancer chemotherapy can suppress production of these white blood cells, leaving patients vulnerable to potentially life-threatening infections and sepsis. G-CSF has thus been widely used with success in cancer patients whose treatment requires high-dose chemotherapy [5]. Furthermore, G-CSF can be used to reinforce the immune system in patients with HIV, pneumonia, diabetic foot infections, leukemia and febrile neutropenia [6-10]. Based on this ample clinical application, the recombinant human G-CSF has been produced in genetically engineered *Escherichia coli *and was approved for use in chemotherapy-induced neutropenia by the U.S Food and Drug Administration in 1991 [11]. It should be pointed out that two types of G-CSF are clinically available: a glycosylated form (lenograstim) which is produced by using the expression in mammalian cells, and nonglycosylated form (filgrastim) which is produced by using the expression in *E. coli*.

Molecular cloning and expression of cDNA for hG-CSF have been described [12,13]. The mature human G-CSF is a 18.8 kDa protein of 174 amino-acid polypeptide chain with two intra-molecular disulphide bonds between residues Cys^36^-Cys^42 ^and Cys^64^-Cys^74 ^and one free cysteine at residue 17 [14]. Native hG-CSF has a single *O*-glycosylation site at Thr^133^, which protects the protein from aggregation but is not crucial for biological activity [15]. The recombinant hG-CSF produced by *E.coli*, has identical biological activity to that of endogenous protein, but differs in that it contains an N-terminal methionine residue and is not glycosylated.

Recent publications describe various protocols of cloning, expression and purification of the rhG-CSF. These protocols involve use of several chromatography columns, high amount of detergents for the purification of G-CSF [16-18] and some of them were not applicable to recombinant G-CSF expressed in *E. coli *as inclusion bodies [19]. In industry, production of biopharmaceuticals employing a simple and cost-efffective process involving fewer steps and yielding high levels of active protein is an essential prerequisite.

Here we describe the cloning of recombinant human granulocyte colony-stimulating factor gene, protein expression in *E. coli *cells, a straightforward purification protocol of the active recombinant protein from inclusion bodies and characterization of rhG-CSF by analytical methods. We believe that the combination of the different experimental strategies presented here provides an efficient protocol that may be useful in the industrial process of rhG-CSF protein production.

## Results and discussion

### Sequence synthesis, cloning and expression

**Figure 1:**
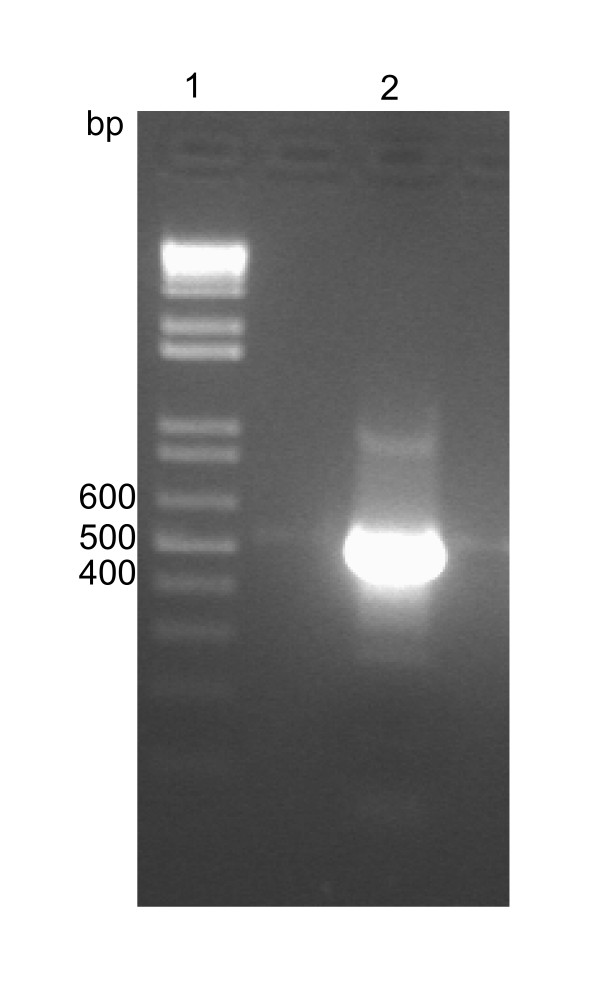
**Agarose gel electrophoresis of PCR product**. Agarose gel (2%) electrophoresis of PCR amplified hG-CSF coding DNA sequence. Lane 1: DNA markers (1 Kb Plus DNA Ladder™, Life Technologies, Gibco BRL); lane 2: PCR amplification of hG-CSF (522 bp).

**Figure 2:**
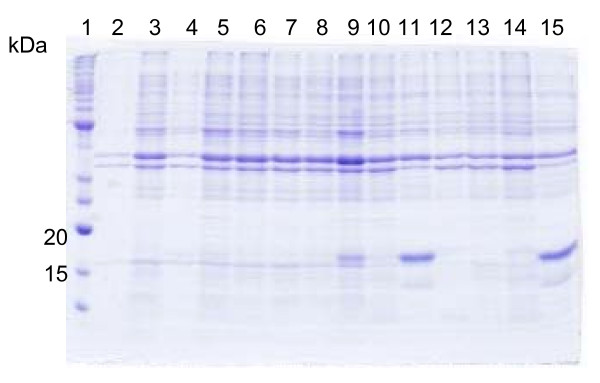
**Analysis of recombinant hG-CSF protein expression by SDS-PAGE**. SDS-PAGE (12%) analysis showing the insoluble fractions of heterologous protein expression in *E. coli *BL21 (DE3) host cells transformed with either pET 23a(+) (control) or pET 23a(+)::hG-CSF. The best expression conditions were 24 h at 37°C without IPTG addition. Lane 1: Molecular mass markers (BenchMark™ Protein Ladder, Invitrogen), lanes 2,6,12: control induced with 0.4 mM IPTG and further growth for 3, 8 and 24 h respectively; lanes 3,7,13: control without induction and further growth for 3, 8 and 24 h respectively; lanes 4,8,10,14: host cells containing the recombinant pET 23a(+)::hG-CSF plasmid induced with 0.4 mM IPTG and further growth for 3, 8, 17 and 24 h respectively; and lanes 5,9,11,15: host cells containing the recombinant pET 23a(+)::hG-CSF plasmid without IPTG induction andd further growth for 3, 8,17 and 24 h respectively. The predicted molecular mass of rhG-CSF is 18.8 kDa.

Synthesis of G-CSF coding DNA sequence was carried out by a method developed in our laboratory, to which a patent has been filed [20]. We used the native sequence of hG-CSF, without making any modification such as replacement of GC rich regions with AT rich regions or replacement of rare codons, as have been reported by others [16,21,22]. A PCR amplification fragment consistent with that expected for hG-CSF (522 bp) was detected on agarose gel (Fig. 1), and its DNA sequence confirmed by automatic sequencing. Recombinant hG-CSF protein was expressed in insoluble form in *E. coli *BL21(DE3) host cells. The best conditions for expression of the rhG-CSF protein in the BL21(DE3) strain were reached at 24 hours in the absence of IPTG induction. In the pET system, target genes are positioned downstream of bacteriophage T7 late promoter. Typically, production hosts contain a prophage (λDE3) encoding the highly processive T7 RNA polymerase under control of the IPTG-inducible *lac*UV5 promoter that would ensure tight control of recombinant gene basal expression [23]. In agreement with the results presented here, high levels of protein expression in the absence of inducer have been shown to occur in the pET system. It has been proposed that protein expression in the absence of induction is a property of *lac*-controlled system when cells approach stationary phase in complex medium and that cyclic AMP, acetate, and low pH are required to achieve high-level expression in the absence of IPTG induction, which may be part of a general cellular response to nutrition limitation [24]. We have previously described this particular feature of the pET system for a number of recombinant proteins [25-27]. The growth rates were the same for *Escherichia coli *BL21(DE3) host cells harboring either pET 23a(+) (control) or the recombinant pET 23a(+)::rhG-CSF plasmid. The absence of IPTG induction to obtain protein expression is thus advantageous since cost is reduced. The presence of the rhG-CSF protein was not observed in the soluble fraction of BL21(DE3) host cells (data not shown). SDS-PAGE showed expression of an insoluble protein with a molecular mass value consistent with that expected for rhG-CSF (18.8 kDa) (Fig. 2). Interestingly, IPTG induction appears to abolish expression of rhG-CSF (Fig. 2, lanes: 4, 8, 10, 14). As pointed out above, T7 RNA polymerase in the pET system is under control of the IPTG-inducible *lac*UV5 promoter, a system that has been used to produce substantial amounts of target protein from a wide variety of genes. However, a few proteins are made in disappointingly small amounts, for reasons that are obvious in some cases and obscure in others. Some likely reasons for obtaining low levels of expression are as follows (pET System Manual, Invitrogen): The target protein itself may interfere with gene expression or with the integrity of the cell, sometimes pulse labeling shows a gradual or rapid decrease in the rate of protein synthesis as target protein accumulates, or sometimes all protein synthesis stops before any target protein can be detected, and occasionally, considerable lysis of a culture is observed. More recently, it has been shown that the predominant contributory factor for decreased production of target proteins from pET plasmids is chromosomal mutation in the prophage of BL21(λDE3) host cells, resulting in decreased levels of functional T7 RNA Polymerase and ensuing reduction in target protein expression [28]. However, the reason for the lack of rhG-CSF protein expression described here is not known yet.

### Purification and characterization of the recombinant G-CSF

The appropriate genetics for soluble protein production has not yet been established and thus prevention of inclusion body formation is essentially a trial-and-error process. Aggregation may be minimized by controlling process parameters such as temperature, reduction of recombinant protein expression rate, adjusting the codon usage, or by co-expression of plasmid-encoded chaperones [29]. However, these strategies do not necessarily result in the same degree of success for different polypeptide chains. Notwithstanding, inclusion bodies can be easily purified and these protein aggregates have also been observed as a source of relatively pure polypeptide [29]. The recovery of biologically active protein from inclusion bodies has several advantages: large amounts of highly enriched protein can be expressed as inclusion bodies; these aggregates are resistant to proteolysis by *E. coli *proteases, allowing high-yield protein production; facilitate production of protein that can be toxic or lethal to the host cell, because inclusion bodies have no biological activity; and, finally, it can be isolated and purified, decreasing the downstream process [30,31]. We have thus employed a strategy to recover active protein from inclusion bodies as described by others [32]. This strategy involves three steps: inclusion body isolation and washing; solubilization of the aggregated protein, and refolding of the solubilized protein. Non-ionic detergents such as Triton × were used to solubilize the bacterial cell wall components that contaminate the inclusion body preparation. EDTA was added to chelate divalent metal ions, which maintain the structural integrity of the cell membrane. A second wash procedure incorporated sodium deoxycholate to remove any residual cell debris particles, especially lipopolysaccharides units that contribute to the unacceptable levels of endotoxins in protein preparations from *E. coli*. The third step using sodium chloride helps displace nucleic acids or any other contaminants that are non-specifically bound to the G-CSF protein in the inclusion bodies by ionic interactions.

**Figure 3:**
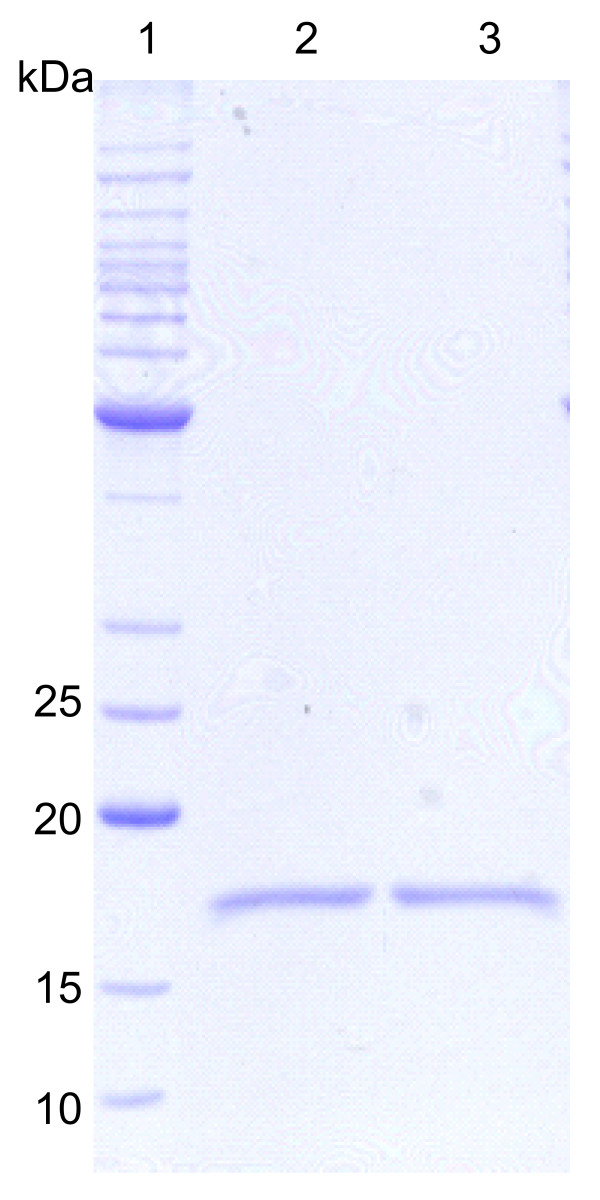
**SDS-PAGE of homogeneous rhG-CSF**. SDS-PAGE (12%) analysis of purified rhG-CSF showing a single protein band. Lane 1: Protein molecular mass markers (BenchMark™ Protein Ladder, Invitrogen), lane 2: homogeneous rhG-CSF (18.8 kDa) after elution from a cation exchange column, lane 3: reference standard. Lanes 2 and 3 contain 1.5 μg of total protein each.

**Figure 4:**
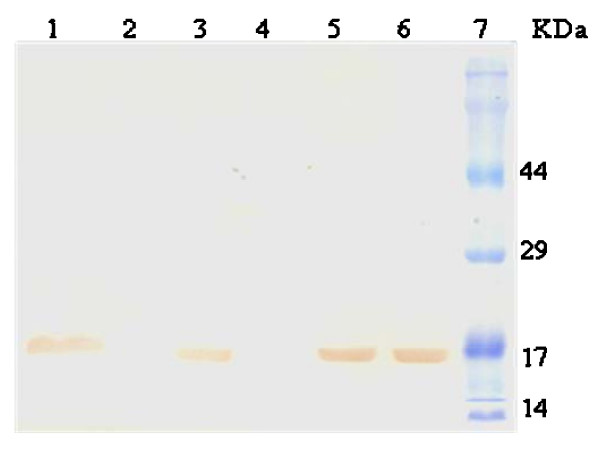
**Western blot of homogeneous rhG-CSF**. A single band was observed for rhG-CSF transferred to a nitrocellulose membrane and detected using a polyclonal antiserum. Lane 1: crude extract of host cells transformed with pET 23a(+)::hG-CSF; lane 2: crude extract of host cells transformed with pET 23a(+) (control); lane 3: insoluble fractions of host cells transformed with pET 23a(+)::hG-CSF; lane 4: insoluble fractions of pET 23a(+); lane 5: homogeneous hG-CSF; lane 6: reference standard; and lane 7: protein molecular mass markers (Cruz Marker™).

rhG-CSF was further purified from inclusion bodies by a cationic exchange column, yielding approximately 3.2 mg of recombinant protein per liter of cell culture. Analysis by SDS-PAGE from the final step of the purification showed a single band of 18.8 kDa, similar to reference standard (Fig. 3). The same general pattern was revealed when the sample shown in Figure 3 was transferred from SDS-PAGE to a nitrocellulose membrane and immunodetected with G-CSF-specific antiserum, confirming a typical profile (Fig. 4) as described by others [33,34]. The first 19 amino-terminal amino acid residues of purified recombinant protein were determined to be MTPLGPASSLPQSFLLKCL by Edman degradation. These results unambiguously demonstrate purification of rhG-CSF protein and that the N-terminal methionine was not removed by post-translational processing.

The solubilization of inclusion bodies pellet method described here employed a combination of small quantity of denaturing agent (2 M urea) and high alkaline pH, different from the majority of the published protocols that used high concentration of solubilizing agent (6 – 8 M). Key to the development of an efficient and economic denaturant-based solubilization step is the determination of the minimum amount of denaturant needed to solubilize the protein and to allow for full bioactivity recovery in the refolding step. In addition, the purification protocol of hG-CSF utilizes only one cation exchange column, which is an improvement upon previous protocols that utilized multiple chromatographic steps [16-18,35]. A method has been described for G-CSF extraction from inclusion bodies produced in *E. coli *that used high amount of detergent and chaotropic agent, and hence additional steps had to be employed to remove these agents, including G-25, CM-cellulose and G-75 chromatographic columns to obtain pure protein [16]. A G-CSF purification protocol has been described that employed immobilized metal affinity chromatography (IMAC) matrix, cation exchange and gel filtration chromatographic steps, the final yields of the protein are not clearly evident [18]. On a commercial scale, reducing the number of protein purification steps is practical and economical because each purification step not only adds to the cost of the final product but also causes successive yield losses of the recombinant protein.

### Physicochemical properties

Characterization of a biological product (which includes the determination of physicochemical properties, biological activity, immunochemical properties, purity and impurities) by appropriate techniques is necessary to confirm an efficient expression and purification protocols. Protein instability encompasses many complicated and interrelated physical and chemical processes. Any of these can occur during the production, isolation and purification of proteins. Chemical degradations that include, at least, deamidation of Asparagine and Glutamine side chain and oxidation of Methionine, as well as dimeric forms and related substances of higher molecular mass, have been recognized to be an important cause of partial loss in activity of therapeutic proteins [36,37]. According to Massiero and coworkers [38], rhG-CSF that undergoes some form of degradation retains only 15% of biological activity. The rhG-CSF here described was analysed by HPLC to detect any of these alterations, including chemical degradation, dimers and related substances of higher molecular mass.

**Figure 5:**
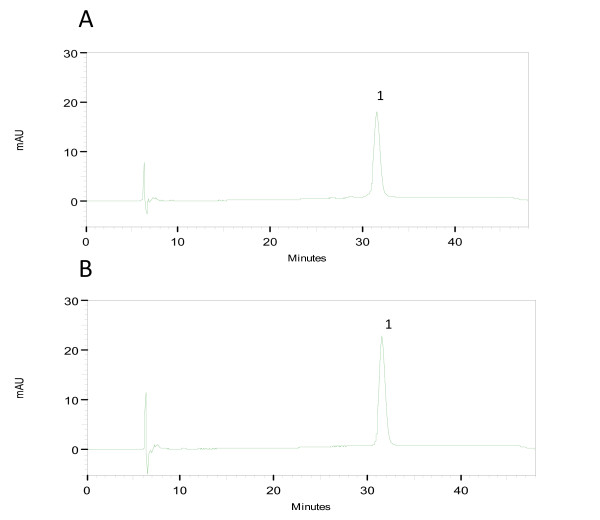
**Reverse-phase HPLC analysis of hG-CSF**. Sulphoxides and deamidates in hG-CSF were analyzed by Reverse-phase HPLC. 200 μg mL^-1 ^of either sample or standard was loaded for analysis. (A): homogeneous hG-CSF, peak 1-main peak. (B): reference standard, peak 1-main peak. Absorbance is in milliabsorbance units (mAU).

**Figure 6:**
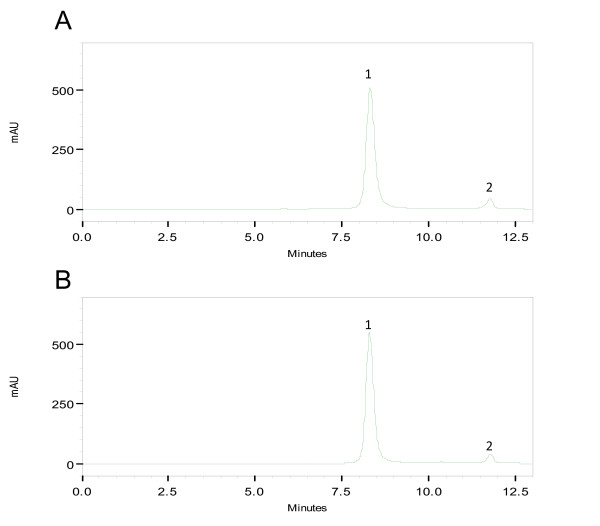
**Size exclusion HPLC analysis of hG-CSF**. Size-exclusion HPLC chromatography was employed to detect dimeric forms of hG-CSF. 200 μg mL^-1 ^of either sample or standard was loaded for analysis. (A): homogeneous hG-CSF, peak 1-monomer and peak 2-excipient. (B): reference standard, peak 1-monomer and peak 2-excipient. Absorbance is in milliabsorbance units (mAU).

Figure 5 shows a typical reverse-phase chromatogram of rhG-CSF demonstrating the resolution of the symmetrical peak that corresponds to hG-CSF with the retention time of 31.6 min similar to standard. The deamidates and sulphoxides were analyzed (Fig. 5) and estimated to be 2.09% and 2.20%, respectively. These values are lower than what is routinely regarded as safe level of 5% for many biopharmaceuticals [39]. Acidic size-exclusion chromatography of rhG-CSF (Fig. 6) demonstrates the presence of a small amount of high molecular weight aggregates (0.26%) and absence of dimers. This level is lower than what is routinely regarded as safe (0.5%) for many biopharmaceuticals [39]. The main peak represents monomers eluted at a retention time similar to that of the standard. Glycosylated G-CSF is freely soluble in phosphate-buffered saline. The absence of carbohydrate however results in a more hydrophobic protein that does not tolerate high salt concentration or neutral pH. It is thus necessary to run size-exclusion chromatography under acidic conditions. At higher pH, recovery of monomers is poor and multimers are irreversibly bound to the resin [40]. The analytical results of recombinant hG-CSF protein reported here are similar to the reference standard. The characterization of rhG-CSF plays a vital role in its development as useful therapeutic agent. Physicochemical techniques can produce information about the structure and composition of this therapeutic protein, but cannot yet predict their biological activity, for which biological assays are required.

### Biological potency evaluation

**Figure 7:**
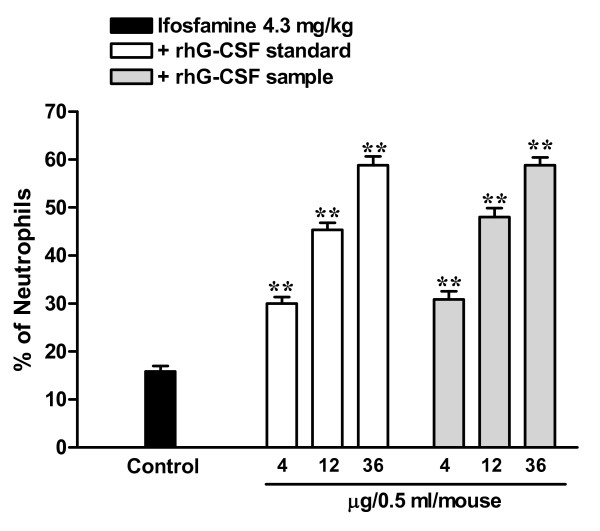
**Biological assay for hG-CSF**. Serum neutrophil counts after treatment with rhG-CSF. Mice in the standard and the sample groups were treated with different doses of rhG-CSF (4, 12, and 36 μg/0.5 ml/mouse). The control group was treated with vehicle (0.1% BSA in PBS). The asterisks represent significant differences in comparison to the control group (** *P*<0.001). *N *= 6 mice per group.

In order to determine the biological potency of rhG-CSF in the present study, we employed an *in vivo *model of neutropenia, by treating mice with a single dose of ifosfamine. The percentage of neutrophils in serum was monitored 6 h after the last rhG-CSF injection or vehicle injection. The data (Fig. 7) clearly demonstrate that treatment with rh-GCSF in the sample group produced a significant and dose-dependent increase of neutrophil counts (*P *<0.001), when compared to the control group (*P *< 0.001). The neutrophil counts in the standard group were not significantly different from that observed in the sample group when comparing the same doses of rhG-CSF (*P *> 0.05, for the three doses).

Statistical analysis by the Finney test [41] revealed a biological activity of 109.4% for the rhG-CSF in the sample group. It appears to be worth pointing out that the potency of Filgrastim (rhG-CSF) itself is not described in any Pharmacopoeia, although values ranging from 90 to 110% have been suggested for biological medicines, according to the guidelines from the European Pharmacopoeia [42]. The data presented clearly indicate that the rhG-CSF produced by the combination of experimental strategies described here can produce biological effects that are comparable or even superior to those obtained with the standard reference rhG-CSF. It is thus tempting to infer that generation of rhG-CSF on a large scale using the protocols described here may represent an interesting option for producing biosimilars.

## Conclusion

In this report, we describe an efficient protocol for cloning, expression and purification of rhG-CSF. The recombinant protein expression in the absence of IPTG may be advantageous since cost is reduced. In addition, the protein purification protocol by liquid chromatography using a single chromatographic step may be a valuable and cost-effective approach to large scale production. The physicochemical, immunological and biological analyses showed that this protocol can be useful to develop therapeutic bioproducts. The data presented here may be of interest to biopharmaceutical companies interested in developing biosimilars, which offer a great opportunity to scientific, economical and industrial growth.

## Methods

### Amplification, cloning and overexpression of hG-CSF coding DNA sequence

Oligonucleotides were manually designed and synthesized, in even numbers, corresponding to the double stranded DNA, in a sequential manner. The G-CSF nucleotide sequence (Accession number NM_000759) of 522 bp, was divided in 12 short sequences of approximately 50 bp each. Oligonucleotides contained overlapping regions of about 10 bases at their 5'- and 3'-ends. The pairs of oligonucleotides were assembled and PCR amplified to yield the hG-CSF coding DNA sequence in a step-wise fashion. PCR-amplified DNA fragments with the expected sizes were detected by 2% agarose gel electrophoresis stained with 0.5 μg mL-1 of Ethidium Bromide, purified and another round of PCR amplification carried out. A thorough description of this method has been presented elsewhere [20]. The primers employed in the present study are given in Table 1. The primers P1 and P12 contained the restriction sites for, respectively, *Nde *I and *Bam *HI. The 522 bp final PCR product was agarose-gel purified and cloned into the pCR^®^-Blunt vector (Invitrogen) and subcloned into the pET 23a(+) expression vector (Novagen) using the *Nde *I and *Bam *HI restriction enzymes (Boehringer Mannheim). The nucleotide sequence of rhG-CSF was determined in order to confirm the identity, integrity, and absence of PCR-introduced mutations in the cloned gene. *Escherichia coli *BL21(DE3) (Novagen) electrocompetent cells were transformed with the recombinant pET 23a(+)::rhG-CSF plasmid and grown on LB agar plates containing 50 μg mL^-1 ^of ampicillin. The protocol for recombinant protein expression described here represents a choice from a number of tests carried out at different experimental conditions. Soluble and insoluble fractions were analyzed by 12% SDS-PAGE [43]. To express and purify the recombinant protein, two liters of 4YT medium (32 g Bacto tryptone, 20 g yeast extract and 5 g NaCl per litre, pH 7.2) containing ampicillin were inoculated with a single colony and grown in shaker flasks for 24 h at 180 rpm and 37°C without IPTG induction. Cells were harvested by centrifugation at 15,900 × *g *for 30 min at 4°C and stored at -20°C.

**Table 1:** Oligonucleotides employed to obtain the coding DNA sequence of hG-CSF*.

P1-5' AA CATATGACCCCCCTGGGCCCTGCCAGCTCCCTGCCCCAGAGCTTCCTGCTC AAGTG 3'
P2-5' GCGCTGCGCCATCGCCCTGGATCTTCCTCACTTGCTCTAAGCACTTGAGCA 3'
P3-5' GGCGCAGCGCTCCAGGAGAAGCTGTGTGCCACCTACAAGCT 3'
P4-5' CCAGAGAGTGTCCGAGCAGCACCAGCTCCTCGGGGTGGCACAGCTTGTAGG 3'
P5-5' CACTCTCTGGGCATCCCCTGGGCTCCCCTGAGCAGCTGCCCCAGCCAGGCCCTG CAG 3'
P6-5' GGTAGAGGAAAAGGCCGCTATGGAGTTGGCTCAAGCAGCCTGCCAGCTGCA GGGCC 3'
P7-5' TTCCTCTACCAGGGGCTCCTGCAGGCCCTGGAAGGGATCTCCCCCGAGTTGGGT C 3'
P8-5' TGGTGGCAAAGTCGGCGACGTCCAGCTGCAGTGTGTCCAAGGTGGGACCCAACT C 3'
P9'-5' TTTGCCACCACCATCTGGCAGCAGATGGAAGAACTGGGAATGGCCCCT GCC CT 3'
P10-5' AAAGCAGAGGCGAAGGCCGGCATGGCACCCTGGGTGGGCTGCAGGGCAGGGG 3'
P11-5' CCTCTGCTTTCCAGCGCCGGGCAGGAGGGGTCCTGGTTGCCTCCCATCTGC AGAGCTTCC 3'
P12-5'AA GGATCCTCAGGGCTGGGCAAGGTGGCGTAGAACGCGGTACGACACCTCCAGGAAGCTCTG 3'

* Primer sequences for construction of hG-CSF. The G-CSF nucleotide sequence (Accession number NM_000759) of 522 bp, was divided into 12 DNA sequences of approximately 50 bp each, containing 10-bp overlapping regions at their 5'- and 3'-ends for adjacent oligonucleotides. The restriction sites, *Nde *I and *Bam *HI, are underlined in primers P1 and P12, respectively.

### Isolation of inclusion bodies

The frozen cell paste was resuspended in lysis buffer (50 mM Tris buffer, at pH 8.0, 1 mM EDTA and 1 mM PMSF) at a pellet:buffer ratio of 1:10 (w/v). The inclusion bodies containing rhG-CSF were recovered from the cells using a French press (Constant Systems LTD) under 137.9 MPa. Prior to centrifugation, a 1:2 dilution of the lysate was carried out to reduce viscosity and to obtain a better yield of inclusion bodies. The resulting lysate solution was centrifuged at 48,000 × *g *for 30 minutes at 4°C to pellet the inclusion bodies. The supernatant was discarded, and the fraction containing the inclusion bodies was subjected to a three-step wash procedure to eliminate endotoxins, proteins and DNA of the host cells. In all steps, the pellet was suspended in the specific buffer at 1:40 (w/v) ratio at room temperature, stirred for 30 min and re-pellet by centrifugation. The first buffer was comprised of: 50 mM Tris, pH 8.0, 5 mM EDTA and 2% Triton ×-100. The composition of the second buffer was 50 mM Tris, pH 8.0, 5 mM EDTA, 1% sodium deoxycholate. In the third wash step, the wash buffer contained 50 mM Tris buffer, pH 8.0, 5 mM EDTA, and 1 M NaCl.

### Solubilization and purification

The protocol of solubilization and denaturation of rhG-CSF in inclusion bodies employed a combination of solubilization agent (2 M urea) and high alkaline pH. The solubilized pellet was stirred for 30 min at room temperature, after diluting the protein to a concentration of 2 mg mL^-1^. Acetic acid was added to bring the pH to a value of 8.0. Refolding of 190 mL of the solubilized protein was carried out by dialysis (MWCO 6,000–8,000 Da) in two steps for 12–16 hours. The first one was carried out against 4 L 50 mM Tris HCl pH 8.0 and the second step against 4 L 25 mM sodium acetate buffer pH 4.5 (buffer A). The purification protocol was performed using the ÄKTA System (GE Healthcare). The soluble protein was purified on a cation exchange column, HiPrep Resource S column (GE Healthcare), preequilibrated with buffer A, and the adsorbed material was washed with buffer A (4 column volumes), eluted with a linear gradient of 0.2 to 0.26 M Tris HCl pH 8.0 (10 column volumes), 0.26 – 1.0 M Tris HCl pH 8.0 (5 column volumes), followed by an isocratic elution with 1.0 M Tris HCl pH 8.0 (5 column volumes) at a flow rate of 1 mL min^-1^. The recombinant hG-CSF eluted between 0.207 – 0.215 M Tris HCl pH 8.0. Protein expression and all protein purification steps were analyzed by 12% SDS-PAGE, and protein concentrations were determined by the method of Bradford *et al*. [44] (Bio-Rad Laboratories).

### N-terminal amino acid sequencing

The N-terminal amino acid residues of homogeneous recombinant hG-CSF were identified by automated Edman degradation sequencing as we have described elsewhere [45].

### Western Blotting

Recombinant rhG-CSF on 12% SDS-PAGE was transferred to a nitrocellulose membrane by electrophoresis at a constant voltage of 70 V in 25 mM Tris pH 8.8, 192 mM glycine, containing methanol 20%, for 1 h using a Trans-blot apparatus (Bio-Rad, USA). The membrane was blocked for 1 h at room temperature by 50 mM sodium phosphate, 150 mM sodium chloride pH 7.0, containing 5% (w/v) dried skim milk powder. The membrane was incubated for 1 h at room temperature with 1.5 μg G-CSF polyclonal antibody (Santa Cruz Biotechnology), in milk-containing phosphate buffered saline as above. The blot was then incubated with a secondary antibody conjugated with horseradish peroxidase (HRPO) (1:1000), and diaminobenzidine (DAB) was used for the development of signal. The size (molecular mass) of the protein was compared to pre-loaded standards (Santa Cruz ™ molecular weight standards).

### Reverse phase liquid chromatography (RP-HPLC) analysis

The sulphoxides and deamidates were analyzed as we have described elsewhere [46]. A HPLC system (Shimadzu, Kyoto, Japan) equipped with an SCL-10A_vp _system controller, LC-10AD_vp _pump, DGU-14A degasser, SIL-10AD_vp _autosampler, and an SPD-M10A_vp _photodiode array (PDA) detector was used. The detector was set at 280 nm and peak areas were integrated automatically by computational analysis, using a Shimadzu Class VP^® ^software program. The experiments were carried out on reversed phase Phenomenex (Torrance, USA) Jupiter C4 column (250 × 4.6 mm I.D, with a pore size of 30 nm). The liquid chromatography system was operated at controlled room temperature (25°C). The elution was performed by a fast gradient at a constant flow rate of 0.5 mL min^-1^. Mobile phase A consisted of water: acetonitrile (90:10 v/v) containing 0.1% trifluoroacetic acid (TFA) and mobile phase B consisted of water: acetonitrile (20:80, v/v) containing 0.1% TFA. The injection volume was 50 μL for both standard and sample. The Filgrastim substance reference used was from NIBISC (National Institute for Biological Standards and Control).

### Size exclusion chromatography (SEC-HPLC) analysis

The detection of dimers and monomers was performed as we have described elsewhere [33]. The HPLC system (Shimadzu, Kyoto, Japan) equipped with an SCL-10A_vp _system controller, LC-10AD_vp _pump, DGU-14A degasser, SIL-10AD_vp _autosampler, and an SPD-M10A_vp _photodiode array (PDA) detector was used. The detector was set at 214 nm and peak areas were integrated automatically by computational analysis, using a Shimadzu Class VP^® ^software program. The experiments were carried out on size exclusion Phenomenex (Torrance, USA) BioSep-SEC 2000 column (300 × 7.8 mm I.D., with a particle size of 5 μm and pore size of 14.5 nm). The HPLC system was operated at room temperature, using mobile phase of phosphoric acid (pH 2.5; 0.1 M) adjusted by the addition of sodium hydroxide (10 N). At the beginning of the run, 50 μL bovine albumin (1 mg mL^-1^) was injected to reduce non-specific adsorption. The injection volume was the same for both, standards and samples, and all determinations were carried out in triplicate. Flow rate was maintained at 1 mL min^-1^. The Filgrastim substance reference used was from NIBISC (National Institute for Biological Standards and Control).

### Biological Assay

Male 7–8 week-old BALB-c mice (19 – 24 g) obtained from the Department of Industrial Pharmacy, Health Science Centre, Universidade Federal de Santa Maria (Brazil), were used in this study. Animals were housed under conditions of optimum light, temperature and humidity (12 h light-dark cycle, 22 ± 2°C, 65% humidity), with food and water provided *ad libitum*. All procedures used in the present study were approved by "Principles of Laboratory Animal Care" from NIH publication No. 85-23 and were approved by the local Animal Ethics Committee with the registration number 19/2005. The number of animals used was the minimum necessary to demonstrate the consistent effects.

The animals were allocated to sample, standard, and control groups in a fully randomized order and identified by color code for assay (6 mice per group). Standard and test sample were diluted to the concentrations of 4, 12, and 36 μg mL^-1^, in phosphate buffered saline (PBS), containing 0.1% bovine serum albumin (BSA). To induce neutropenia, all mice received a single dose of ifosfamine (4.3 mg/0.5 mL per animal) by intraperitoneal route, on day 0, according to the methodology we have described elsewhere [46]. Animals in the treated groups received multiple injections of 0.5 mL rhG-CSF (standard or sample groups), from day 1 to day 4. The control group was treated with vehicle (0.1% BSA in PBS) at the same schedule of administration. Six hours after the last rhG-CSF injection or vehicle, peripheral blood was collected from the orbital venus sinus. Smears were prepared on glass slides and stained by the May-Grünwald-Giemsa method, and the white cells were counted and expressed as the total number of neutrophils. The Filgrastim standard used was from NIBISC (National Institute for Biological Standards and Control).

### Statistical analysis

The results (% of neutrophils) are presented as the mean ± S.D. of 6 animals. Statistical comparison of data was performed by one-way analysis of variance (ANOVA) followed by Bonferroni's post-test, by means of GraphPad Prism Program (Version 4.0). *P*-values less than 0.05 (*P *< 0.05 or less) were considered significant.

For determining the possible differences in the biological activity between the standard and the sample groups, the test described by Finney [41] was adopted, through the parallel line method (3 × 3), using a PLA 1.2 program (Stegmann Systemberatung, Rodgau, Germany). Analysis of variance was performed for each assay, and the assumption of linearity and parallelism of the log dose-log response lines was tested (*P *< 0.05). Statistical weights were computed as the reciprocal of the variance of the log potency. Estimates of log potency were examined for heterogeneity using a χ^2 ^test (*P *= 0.05) and were combined as weight geometric means of homogeneous estimates (*P *> 0.05) [47].

## Abbreviations

BSA: bovine serum albumin, DAB: diaminobenzidine, HRPO: horseradish peroxidase, IPTG: isopropyl-β-D-thiogalactopyranoside, kDa: kilodalton, PBS: phosphate buffered saline containing, PCR: polymerase chain reaction, PMSF: phenyl methyl sulfonyl fluoride, rhG-CSF: recombinant human granulocyte colony stimulating factor, RP-HPLC: reverse phase liquid chromatography, SDS-PAGE: sodium dodecyl sulfate-polyacrylamide gel eletrophoresis, SEC-HPLC: Size exclusion liquid chromatography.

## Competing interests

The author(s) declare that they have no competing interests.

## Authors' contributions

ALSV performed most of the experiments and drafted the manuscript. GR supervised cloning, expression and purification experiments and helped draft the manuscript. MSP carried N-terminal protein sequencing analysis. JMC participated in the analysis and interpretation of protein chemistry results. SLD supervised physicochemical and biological activity analysis and helped draft the manuscript. The study was conceived and coordinated by DS and LB, who have also revised the manuscript. All authors read and approved the final manuscript.
